# Fatty Acid Synthase Contributes to Restimulation-Induced Cell Death of Human CD4 T Cells

**DOI:** 10.3389/fmolb.2019.00106

**Published:** 2019-10-15

**Authors:** Kelsey Voss, Christopher R. Luthers, Katherine Pohida, Andrew L. Snow

**Affiliations:** Department of Pharmacology & Molecular Therapeutics, Uniformed Services University of the Health Sciences, Bethesda, MD, United States

**Keywords:** T cell, RICD, apoptosis, metabolism, fatty acid synthase, glycolysis, FAS, FAS ligand

## Abstract

Restimulation-induced cell death (RICD) is an apoptotic pathway triggered in activated effector T cells after T cell receptor (TCR) re-engagement. RICD operates at the peak of the immune response to ensure T cell expansion remains in check to maintain immune homeostasis. Understanding the biochemical regulation of RICD sensitivity may provide strategies for tuning the magnitude of an effector T cell response. Metabolic reprogramming in activated T cells is not only critical for T cell differentiation and effector functions, but also influences apoptosis sensitivity. We previously demonstrated that aerobic glycolysis correlates with optimum RICD sensitivity in human effector CD8 T cells. However, metabolic programming in CD4 T cells has not been investigated in this context. We employed a pharmacological approach to explore the effects of fatty acid and glycolytic metabolism on RICD sensitivity in primary human CD4 T cells. Blockade of fatty acid synthase (FASN) with the compound C75 significantly protected CD4 effector T cells from RICD, suggesting that fatty acid biosynthesis contributes to RICD sensitivity. Interestingly, sphingolipid synthesis and fatty acid oxidation (FAO) were dispensable for RICD. Disruption of glycolysis did not protect CD4 T cells from RICD unless glyceraldehyde-3-phosphate dehydrogenase (GAPDH) enzymatic activity was targeted specifically, highlighting important differences in the metabolic control of RICD in effector CD4 vs. CD8 T cell populations. Moreover, C75 treatment protected effector CD4 T cells derived from naïve, effector memory, and central memory T cell subsets. Decreased RICD in C75-treated CD4 T cells correlated with markedly reduced FAS ligand (FASL) induction and a Th2-skewed phenotype, consistent with RICD-resistant CD4 T cells. These findings highlight FASN as a critical metabolic potentiator of RICD in human effector CD4 T cells.

## Introduction

Dynamic metabolic reprogramming propels the rapid proliferation of activated T cells, ensuring clonal expansion is robust enough to eliminate pathogens or tumor cells. However, T cell expansion must also be curtailed efficiently to maintain homeostasis and preserve a small pool of long-lived memory cells for future defense. Upon initial antigen encounter with proper co-stimulation, activated T cells ramp up their anabolic metabolism resulting in increased Krebs cycle activity, oxidative phosphorylation (OXPHOS), and aerobic glycolysis (Pearce, 2010). The rapid increase in glycolysis is proportionately greater than other metabolic pathways despite a lower net ATP gain. As in tumor cells, this “Warburg effect” is presumed to support the production of energy and macromolecular intermediates that enable the swift cell division (Ghesquiere et al., 2014). However, CD4 T cells also rely on the uptake of amino acids like glutamine, arginine and leucine to fuel proliferation, whereas glycolysis may be more important for differentiation and effector functions (e.g., cytokine production) (Buck et al., 2015; Palmer et al., 2015). Inhibitors of metabolic pathways (e.g., Krebs cycle inhibitor LW6) have therefore shown the potential to be potent immunosuppressive drugs (Eleftheriadis et al., 2017).

Programmed cell death is critical for preserving adaptive immune homeostasis, as it is the essential process for disposal of excess T cells reacting to either foreign or self-antigens. T cells are specifically programmed to die by apoptosis at different phases throughout a healthy T cell response (Green et al., 2003; Brenner et al., 2008; Li et al., 2017). Restimulation-induced cell death (RICD) occurs at the peak of T cell expansion when previously activated T cells are restimulated through the T cell receptor (TCR) in the presence of interleukin 2 (IL-2). After antigen is cleared, most effector T cells are culled through cytokine withdrawal-induced death (CWID), an intrinsic apoptosis pathway triggered by the loss of IL-2 signaling (Snow et al., 2010). RICD has particular relevance to immune homeostasis during viral infections. Notably, X-linked lymphoproliferative disease (XLP-1) patients with null mutations in the small signaling adaptor SLAM-associated protein (SAP) experience life-threatening accumulation of RICD-resistant T cells during certain viral infections (Sharifi et al., 2004; Bassiri et al., 2008; Snow et al., 2009). Therefore, strategies that restore or boost RICD sensitivity have the potential to cull excessive T cell responses and limit immunopathology in those circumstances (Ruffo et al., 2016). Conversely, novel therapies that reduce RICD could be employed to boost insufficient effector T cell responses in response to infection, vaccination, or tumorigenesis.

Recent work indicates that cellular metabolism is intricately associated with apoptosis sensitivity (Voss et al., 2017). In CD8 T cells, we showed that catabolic metabolism via autophagy could delay CWID of effector T cells derived from the central vs. effector memory pool (Larsen et al., 2017). Interestingly, we also found that maximal RICD sensitivity in human CD8 effector T cells is linked to elevated glycolysis (Larsen et al., 2017). Glycolysis specifically facilitates TCR-induced FAS ligand (FASL) upregulation, which contributes to RICD by engaging the extrinsic apoptosis pathway through the FAS death receptor. In this manner, glycolytic metabolism may effectively calibrate an optimal effector CD8 T cell response by both driving and constraining T cell expansion when antigen is still abundant. Interestingly, viruses often intervene with glycolytic metabolism of T cells to support their own replication, highlighting an important relationship between host cell metabolism and viral infections (Pallett et al., 2019).

Metabolic contributions to RICD sensitivity have not been assessed previously in human CD4 T cells. Therefore, we sought to investigate the relative contributions of fatty acid and glycolytic metabolism on the sensitivity of human CD4 effector T cells to RICD sensitivity. Here we show that CD4 T cells are significantly rescued from RICD upon blockade of fatty acid synthase (FASN), which reduced FASL induction upon TCR restimulation and shifted T cells to a Th2-like, RICD-resistant phenotype. These data provide compelling evidence that pharmacological inhibitors of metabolic checkpoints alter apoptosis sensitivity in T cell subsets differently, with ramifications for their use in cancer therapy and in novel approaches designed to tune T cell responses.

## Results

### Pharmacological Inhibitors of Fatty Acid Metabolism Differentially Impact RICD Sensitivity

**Figure 1:**
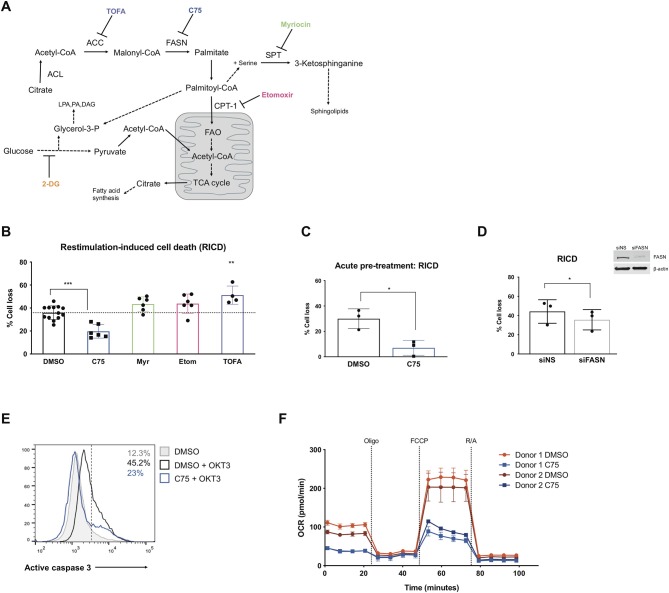
Inhibiting fatty acid synthase decreases RICD in human CD4 T cells. CD4 T cells were activated and expanded in culture for ~12 days. **(A)** Schematic pathway diagram indicating targets of pharmacological inhibitors of metabolism: TOFA, C75, myriocin, and etomoxir. ACL, ATP-citrate lyase; ACC, acetyl-CoA carboxylase; FASN, fatty acid synthase; SPT, serine palmitoyltransferase; LPA, lysophosphatidic acid; PA, phosphatidic acid; DAG, diacyl glycerol. **(B)** CD4 T cells were pre-treated with inhibitors overnight and then restimulated with 100 ng/ml OKT3 for 24 h. RICD was measured by propidium iodide (PI) staining. Each data point represents the average % cell loss from an individual donor. One-way ANOVA with Dunnett's multiple comparisons test: ^***^p < 0.0001, ^**^p = 0.0044. **(C)** CD4 T cells were treated with C75 or DMSO for 1 h prior to OKT3 stimulation. RICD assays were conducted as above. *t*-test ^*^p = 0.0154 **(D)** CD4 T cells were transfected with siRNA against fatty acid synthase (siFASN) or a non-specific scramble control (siNS). T cells were restimulated with 100 ng/ml OKT3 4 days post-transfection and RICD was measured by PI staining 24 h later. Knockdowns were verified by immunoblot (right). Paired *t*-test ^*^p = 0.0145 **(E)** T cells were treated with DMSO or C75 for 1 h and then restimulated with OKT3 for 24 h. Active caspase 3 staining was quantified by flow cytometry. Figure shows one representative experiment (*N* = 3). **(F)** CD4 T cells from two healthy donors were pre-treated overnight with either C75 or a DMSO control on day 12 post-activation. Oxygen consumption rate (OCR) was measured over time on the Seahorse XFe96. DMSO samples are indicated with circles and C75 samples by squares. Oligomycin A = Oligo, trifluoromethoxy carbonylcyanide phenylhydrazone = FCCP, rotenone + antimycin A = R/A.

In order to investigate how metabolic programs affect RICD sensitivity in human CD4 T cells, we purified and activated primary CD4 T cells from healthy human donors *in vitro*. Purity of CD4 T cell isolations was ~95% (Supplemental Figure 1A). After 12 days of expansion in IL-2, effector CD4 T cells were treated with various pharmacological inhibitors of metabolic pathways (Figure 1A). C75 (4-methylene-2-octyl-5-oxotetra-hydrofuran-3-carboxylic acid) inhibits FASN, a multi-enzyme protein that catalyzes the synthesis of palmitate from acetyl-CoA and malonyl-CoA. 5-tetradecyloxy-2-furoic acid (TOFA) also inhibits fatty acid metabolism, but instead acts by blocking the first step of fatty acid biosynthesis: the synthesis of malonyl-CoA by acetyl coenzyme carboxylase (ACC). Etomoxir inhibits carnitine palmitoyltransferase 1 (CPT1), an enzyme that transports fatty acids into the mitochondria for β-oxidation—a pathway heavily utilized by long-lived memory T cells. To interrogate sphingolipid metabolism, we utilized myriocin—a potent inhibitor of serine palmitoyltransferase (SPT), which catalyzes the first step in sphingosine biosynthesis. Finally, we reduced glycolysis using 2-deoxy-D-glucose (2-DG), which competes for the phosphorylation of glucose by hexokinase (HK).

Activated CD4 T cells pre-treated with inhibitors of fatty acid metabolism were restimulated through the TCR and assessed for death by RICD the next day as previously described (Katz and Snow, 2013). T cells pre-treated with C75 demonstrated a marked reduction in RICD sensitivity in every donor (Figure 1B), suggesting that fatty acid synthesis contributes to RICD. This decrease in death sensitivity was also observed with an acute pre-treatment of C75 added just prior to restimulation (Figure 1C). We noted that C75 treatment did not have a significant impact on fatty acid translocase (CD36) expression, which was very low on effector CD4 T cells (Supplemental Figure 1B). Moreover, C75-induced protection from RICD was specifically linked to reduced apoptosis, as shown by a decreased proportion of T cells with active caspase 3 following TCR restimulation (Figure 1E).

Myriocin was previously reported to block activation-induced cell death in T cell hybridomas by interfering with FAS-induced caspase activation (Solomon et al., 2003). Sphingolipid metabolism has also been associated with increased apoptosis sensitivity via sphingolipid products cooperating with BAX and BAK to destabilize mitochondrial outer-membrane potential (MOMP) (Solomon et al., 2003). Despite these reports, we observed that myriocin treatment in primary human CD4 T cells did not impact RICD sensitivity (Figure 1B). Interestingly, etomoxir treatment also had little impact on RICD sensitivity, suggesting that fatty acid oxidation (FAO) is dispensable for death sensitivity in effector CD4 T cells (Figure 1B). Given that high concentrations of etomoxir have reported off target effects in T cells (Raud et al., 2018), we tested RICD under various doses. While we saw no change in RICD sensitivity at 10 μM, there was a slight but significant increase in RICD at 5 μM (Supplemental Figure 1C). Interestingly, increased RICD sensitivity was not seen at 1 μM etomoxir treatment, highlighting the importance of assessing multiple doses of this inhibitor on downstream phenotypes.

Although inhibition of FASN with C75 treatment greatly reduced RICD sensitivity, blockade of ACC with TOFA did not reduce RICD (Figure 1B). In fact, TOFA treatment resulted in a slight increase in RICD sensitivity. Given the differential effects of pharmacological inhibition of enzymes in the same biosynthetic pathway, we specifically targeted FASN expression with small interfering RNA (siRNA). A knockdown of FASN expression resulted in a decrease in RICD sensitivity (Figure 1D), consistent with C75 treatment (Figures 1B,C). These results collectively suggest that fatty acid synthesis by FASN specifically influences RICD sensitivity in CD4 T cells.

We noted that although statistically significant, the reduction in RICD by FASN knockdown (Figure 1D) was not as robust as C75 treatment (Figure 1B), which could be due in part to suboptimal knockdown efficiency. However, C75 could have off-target interactions that provide additional protection from RICD. For example, biological targets identified in human liver cancer cells using a library of tagged C75 analogs revealed previously unknown putative C75 targets, including protein disulfide-isomerase A3 (PDIA3), transferrin receptor (TRFC), and glyceraldehyde-3-phosphate dehydrogenase (GAPDH) (Cheng et al., 2014). Given these possibilities, we analyzed the general bioenergetic state of T cells pre-treated with C75 or a DMSO control (Figure 1F). The oxygen consumption rate (OCR) of CD4 T cells from 2 different donors was measured during an Agilent Seahorse XF Mito Stress Test in order to measure mitochondrial function. In both donors, CD4 T cells treated with C75 showed a significantly different profile of mitochondrial function and oxygen consumption (Figure 1F). The basal respiration rate before oligomycin A (oligo) injection was lower in C75-treated cells. Additionally, the maximal mitochondrial respiration of C75-treated cells was impaired compared to the DMSO-treated controls after FCCP injection (Figure 1F), demonstrating a distinct profile of mitochondrial respiration.

We also wondered whether blockade of FASN in CD8 T cells would reduce RICD, given the great reduction in CD4 T cells. Indeed, although CD8 T cells are generally more sensitive to RICD than CD4 T cells, C75 treatment also significantly reduced death (Supplemental Figure 2A), whereas other fatty acid metabolism inhibitors had no effect. This was also the case with acute pre-treatment of C75 (vs. overnight treatment) at multiple doses of OKT3 restimulation (Supplemental Figure 2B).

### C75 Treatment Results in Decreased ATP Production and Glycolysis

**Figure 2:**
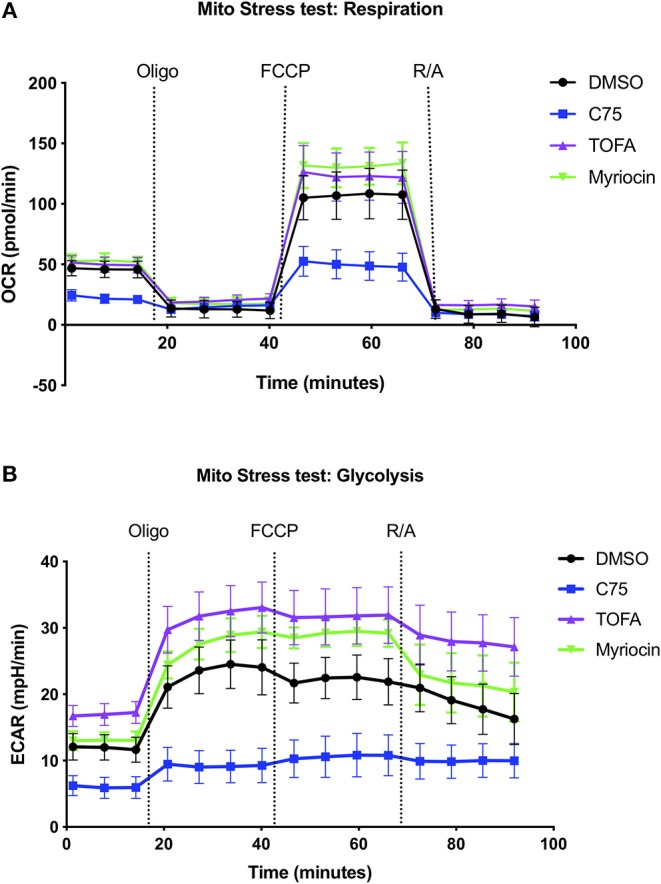
Inhibiting fatty acid metabolism alters CD4 T cell respiration and glycolysis. At 12 days post-activation, CD4 effector T cells from healthy donors were treated with various inhibitors overnight. **(A)** The oxygen consumption rate (OCR) and **(B)** extracellular acidification rate (ECAR) were measured over time using the Seahorse XFe96 analyzer. Figure shows one representative experiment of which similar results were obtained in at least four different donors.

Given the robust differences in RICD sensitivity and respiration of C75-treated cells, we sought to further characterize metabolic changes in CD4 effector T cells upon C75 treatment in comparison to other inhibitors that did not impact RICD sensitivity (Figure 1B). To this end, we expanded our bioenergetic analyses to T cells pre-treated with C75, myriocin, TOFA, or a DMSO control to investigate whether changes in particular bioenergetic features could be specifically associated with changes in RICD sensitivity. C75 treatment reduced basal respiration compared to all other treatment conditions (Figure 2A). The maximal mitochondrial respiration after FCCP injection was severely impaired in C75-treated T cells, consistent with the previous experiment (Figure 1F). In contrast, the maximal respiration rate of TOFA- and myriocin-treated cells was higher than the DMSO control (Figure 2A). We also examined the extracellular acidification rate (ECAR) as a read-out for glycolytic flux (Figure 2B). Unexpectedly, C75-treated cells were significantly lower in glycolytic activity compared to all other treatment groups (Figure 2B), and TOFA treatment resulted in a slight increase in glycolysis, correlating with a slight increase in RICD sensitivity (Figure 1B). Therefore, we posited that C75 treatment could be protecting CD4 T cells from RICD by altering basal mitochondrial respiration and/or by lowering glycolysis, which we have previously linked to RICD sensitivity in CD8 T cells (Larsen et al., 2017).

**Figure 3:**
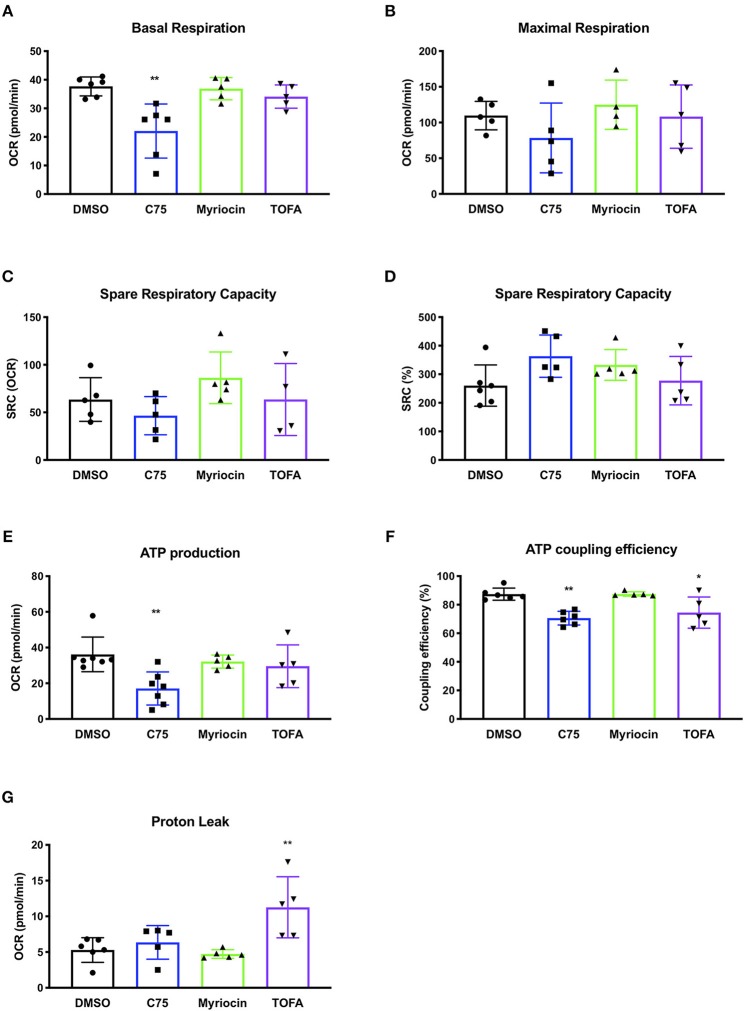
Changes in mitochondrial bioenergetic functions in response to fatty acid metabolism inhibitors in human CD4 effector T cells. CD4 T cells were treated with various inhibitors overnight on day 12 post-activation. Mito Stress Tests were conducted on the Seahorse XFe96 (Agilent). Each data point represents an individual donor. **(A)** Basal respiration = (last rate measurement before first injection – non-mitochondrial respiration rate). Repeated measures (RM) one-way ANOVA with Dunnett's multiple comparisons test ^**^p = 0.003. **(B)** Maximal respiration rate = (maximum rate measurement after FCCP injection – non-mitochondrial respiration). **(C)** Spare respiratory capacity (SRC) = (maximal respiration – basal respiration). **(D)** SRC as a percentage of total capacity. **(E)** ATP production = (last rate measured before oligomycin injection – minimum rate measurement after oligomycin injection). One-way ANOVA with Dunnett's multiple comparisons test ^***^p = 0.0005. **(F)** ATP coupling efficiency = [(ATP production rate)/(basal respiration rate) × 100]. RM one-way ANOVA with Dunnett's multiple comparisons test ^**^p = 0.0051, ^*^p = 0.0426. **(G)** Proton leak = (minimum rate measurement after oligomycin injection – non-mitochondrial respiration). RM one-way ANOVA with Dunnett's multiple comparisons test ^**^p = 0.0079.

We repeated these metabolic flux analyses in CD4 T cells from multiple donors to expand our findings and look for a bioenergetic pattern that correlated with RICD resistance. C75 treatment significantly reduced the basal oxygen respiration rate (last rate measurement before first injection – non-mitochondrial respiration rate) compared to all other treatment groups (Figure 3A). The maximal respiration rate (maximum rate measurement after FCCP injection – non-mitochondrial respiration) was also reduced, although not statistically significant (Figure 3B). The spare respiratory capacity (SRC) (maximal respiration – basal respiration) was somewhat reduced in C75-treated cells (Figure 3C), but not when SRC was calculated a percentage (Figure 3D). The most significantly impacted parameter was ATP production (last rate measured before oligomycin injection – minimum rate measurement after oligomycin injection), which was much lower in C75 treated cells compared to all other treatment groups (Figure 3E). The ATP coupling efficiency [(ATP production rate)/(basal respiration rate) × 100] was also significantly reduced in C75-treated cells, although TOFA treatment also lowered ATP coupling efficiency (Figure 3F). Only TOFA-treated cells tended to show an increase in proton leak (minimum rate measurement after oligomycin injection – non-mitochondrial respiration) (Figure 3G), which can be considered a sign of mitochondrial damage. Importantly, the severe reduction in mitochondrial activity in C75-treated cells could not be explained by a reduction in overall mitochondrial mass; overnight treatment with all inhibitors did not alter mitochondrial mass (Supplemental Figure 1D). Collectively, our bioenergetics analyses suggest that decreased ATP production and basal respiration could correlate with more resistance to RICD, whereas increased proton leak could be associated with increased susceptibility to RICD.

### Differential Effects of Blocking Glycolysis on RICD Sensitivity in CD4 T Cells

**Figure 4:**
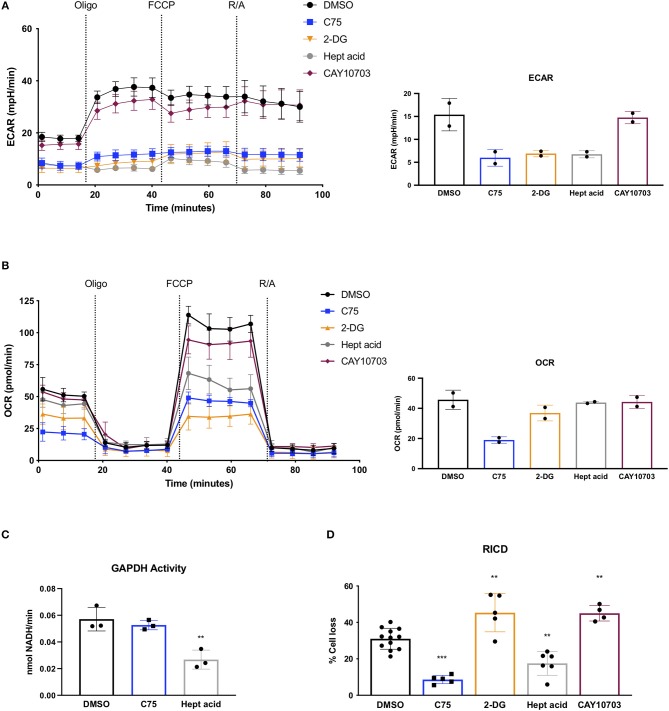
Effects of C75 and glycolysis inhibitors on RICD and GAPDH activity. CD4 T cells were treated with various inhibitors overnight on day 12 post-activation. **(A)** Extracellular acidification rate (ECAR) was measured over time using the Seahorse XFe96 analyzer (Agilent). Figure shows one representative experiment of which similar results were obtained in two different donors. Basal ECAR from both donors is shown (right). **(B)** OCR over time was measured as in **(A)** from 2 different donors. Basal OCR from both donors is shown (right). **(C)** GAPDH activity was measured in CD4 T cells that had been treated overnight with DMSO, C75, or heptelidic acid. Each data point represents the average triplicate value from an individual donor. RM one-way ANOVA with Dunnett's multiple comparisons test ^**^p = 0.003. **(D)** CD4 T cells were treated with various inhibitors 1 h before restimulation with 100 ng/ml OKT3. RM one-way ANOVA with Dunnett's multiple comparisons test ^***^p < 0.0001, ^**^p = 0.001, ^**^p = 0.009, ^**^p = 0.0028.

Given the unexpected result of C75 treatment on lowering glycolysis, we compared C75 treatment to other glycolytic inhibitors. Upon further ECAR analysis, C75 lead to a decrease in glycolysis comparable to the competitive glucose analog 2-DG (Figure 4A). The largest decrease in glycolytic flux was observed by heptelidic acid treatment (Figure 4A), which specifically inhibits the catalytic site of GAPDH, the third enzymatic step in the glycolysis pathway. CAY10703, a stable derivative of dichloroacetate, is also considered an inhibitor of glycolysis by targeting pyruvate dehydrogenase kinase (PDHK). However, unlike C75 or 2-DG, overnight pre-treatment of CAY10703 did not significantly lower ECAR (Figure 4A). Interestingly, only C75 and 2-DG treatment lowered basal OCR (Figure 4B), suggesting that heptelidic acid may be a superior means of specifically inhibiting glycolysis without affecting oxidative metabolism.

Since C75 was inhibiting glycolysis as effectively as the standard glycolytic inhibitor 2-DG, we asked whether C75 was interfering with GAPDH activity. Indeed, C75 was reported to bind to GAPDH non-specifically (Cheng et al., 2014). However, T cells treated with C75 overnight showed no decrease in GAPDH activity (Figure 4C), suggesting that the putative interaction between C75 and GAPDH does not impair its' enzymatic function. Importantly, heptelidic acid demonstrated a significant decrease in GAPDH activity as expected (Figure 4C). Preliminary experiments from 2 donors showed no significant decrease in GAPDH activity upon 2-DG treatment overnight (data not shown).

To directly test whether differential glycolytic flux impacts RICD sensitivity in CD4 T cells, T cells were pre-treated for 1 h with C75, 2-DG, heptelidic acid, or CAY10703 and restimulated overnight to measure RICD. Although C75 treatment reliably decreased RICD sensitivity, 2-DG and CAY10703 treatment surprisingly triggered more RICD in CD4 effector T cells (Figure 4D). Moreover, heptelidic acid treatment decreased RICD sensitivity, but not to the extent of C75 treatment (Figure 4D). Taken together, these results suggest that C75 likely modulates RICD independently of glycolytic disruption, but also highlight a complicated relationship between glycolysis and RICD in primary T cells that requires further study.

### Effect of Metabolic Inhibitors on Molecular Determinants of RICD Sensitivity

**Figure 5:**
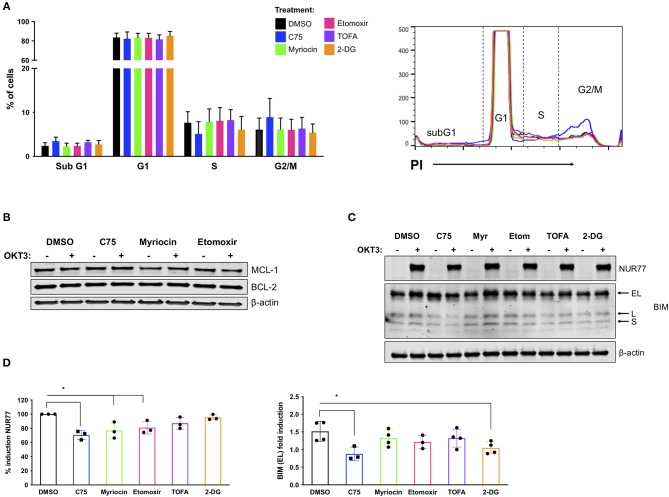
C75 treatment impacts cell cycle status and reduces pro-apoptotic molecules associated with RICD. CD4 T cells from healthy donors were treated with various inhibitors overnight on day 12 post-activation. **(A)** PI cell cycle analysis was conducted on samples from 4 different donors. Figure shows the combination of the percentage of cells in each cell cycle stage from all 4 donors. **(B)** Anti-apoptotic proteins MCL-1 and BCL-2 were analyzed in whole cell lysates of CD4 T cells pre-treated overnight with various inhibitors. T cells were either left unstimulated or were restimulated with 200 ng/ml OKT3 for 4 h. Results are representative of 3 independent experiments. **(C)** To analyze pro-apoptotic BIM and NUR77 expression, T cells were restimulated with 200 ng/ml OKT3 for 4 h. Whole cell lysates were subjected to immunoblot analysis with β-actin as a loading control. BIM detection consists of 3 transcripts: extra-long (EL), long (L), and short (S). Figure is representative of 3 independent experiments. **(D)** NUR77 and BIM EL induction upon restimulation as in **(C)** were quantified after normalization to β-actin. The relative amount of NUR77 or BIM EL was compared to the relative amount in the DMSO control for that experiment and plotted as an individual data point. **(D)** NUR77; RM one-way ANOVA: C75 ^*^p = 0.0017, Myriocin ^*^p = 0.0102, Etomoxir ^*^p = 0.0334. BIM EL; RM one-way ANOVA: C75 ^*^p = 0.0088, 2-DG ^*^p = 0.0402.

To delineate a mechanism by which C75 decreased RICD sensitivity in CD4 T cells, we investigated the effect of the various inhibitors on cell cycle status and other parameters known to influence RICD sensitivity. CD4 T cells from 4 separate donors were treated with inhibitors overnight and subjected to cell cycle analysis the next day (Figure 5A). C75 treatment induced a modest decrease in the percentage of cells cycling in S phase compared to all other treatment groups; progression through S phase is a known prerequisite for RICD (Boehme and Lenardo, 1993). We also observed a concomitant slight increase in the percentage of cells in G2/M with C75 exposure (Figure 5A). However, to what extent these modest changes in cell cycle status may contribute to the large decrease in RICD sensitivity demonstrated by C75 treated cells is not clear.

RICD sensitivity is tuned by a balance of pro- and anti- apoptotic molecules, including BCL-2 family members that control intrinsic apoptosis through the mitochondria (Snow et al., 2008). We did not detect any notable differences in anti-apoptotic family members MCL-1 or BCL-2 in any treatment groups (Figure 5B), either at baseline or after TCR restimulation. However, there were notable decreases in the pro-apoptotic proteins NUR77 and BIM in C75-treated cells (Figure 5C). NUR77 induction is a sensitive readout of TCR stimulation, and has previously been associated with RICD (Cheng et al., 1997; Toth et al., 2001). Upon quantification, a decrease in NUR77 expression was significant in C75, myriocin, and etomoxir treated cells (Figure 5D). BIM induction has similarly been associated with RICD sensitivity (Snow et al., 2008), and we noted a significant decrease in both C75 and 2-DG treated T cells based on quantification of the readily detectable extra-long (EL) isoform (Figure 5D). Hence although a reduction in NUR77 and BIM expression correlates with reduced RICD in C75 treated cells, these effects likely do not explain the differences in RICD sensitivity specifically between C75 and other metabolic inhibitors tested (Figure 1B).

**Figure 6:**
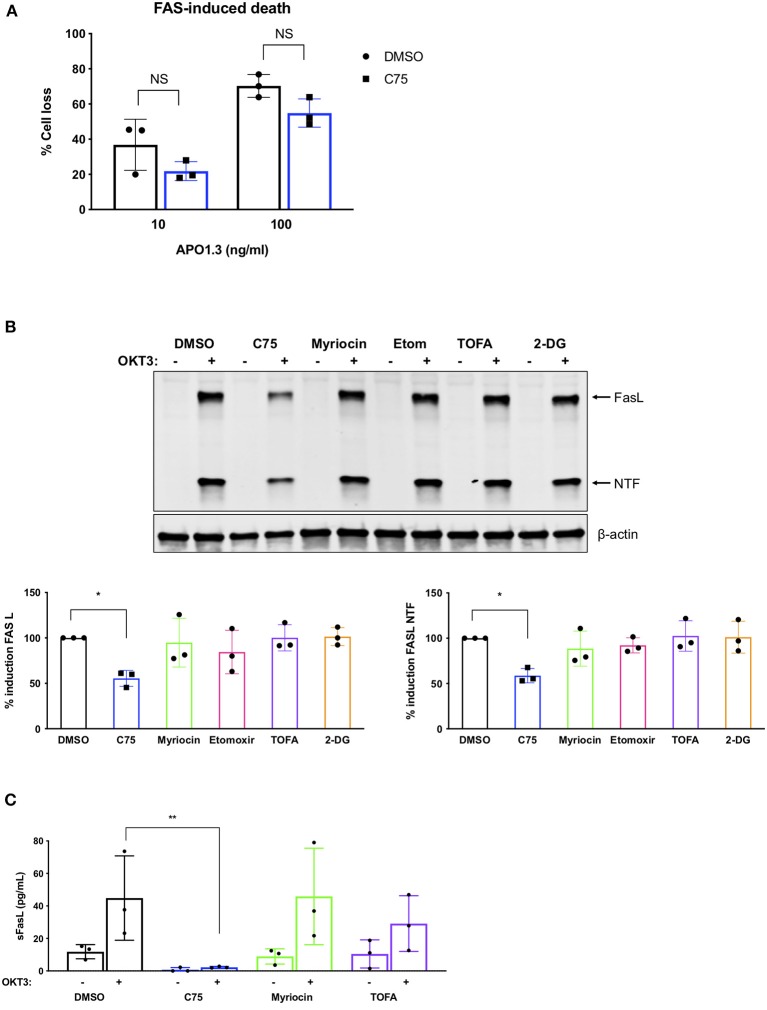
C75 significantly reduces the induction of FAS ligand protein expression. CD4 T cells from healthy donors were treated with various inhibitors overnight on day 11 or 12 post-activation. **(A)** Death by FAS ligation was measured by propidium iodide staining 24 h after APO1.3 stimulation + Protein A. NS = not significant by two-way ANOVA. **(B)** Pre-treated T cells were restimulated with 200 ng/ml OKT3 for 4 h. Whole cell lysates were subjected to immunoblot analysis with β-actin as a loading control. FAS ligand (FASL) induction was quantified as well as the cytoplasmic N-terminal fragment (NTF) cleavage product. One-way ANOVA with Dunnett's multiple comparisons test ^*^p = 0.0263, NTF ^*^p = 0.0115. **(C)** Pre-treated T cells were restimulated with 200 ng/ml OKT3 for 4 h. Supernatants were then subjected to ELISA for soluble FASL (sFASL) quantification. One-way ANOVA with Sidak's multiple comparisons test ^**^p = 0.0056.

We next analyzed TCR restimulation-induced upregulation of FASL, which is considered to be the key pro-apoptotic driver of RICD in CD4 T cells via ligation of the FAS death receptor. Although C75 did not significantly alter apoptosis triggered by direct antibody-dependent crosslinking of FAS (Figure 6A), we did observe a substantial decrease in the upregulation of FASL protein with C75 alone relative to other inhibitors (Figure 6B). This unique C75-dependent decrease in FASL expression was significant based on quantification of both full length FASL and the cytoplasmic N-terminal fragment, generated by cleavage of FASL at the plasma membrane (Figure 6B). Indeed, TCR-triggered release of soluble FASL (sFASL) from C75 treated CD4 T cells was almost undetectable relative to other inhibitors tested (Figure 6C). Therefore, C75 treatment may protect T cells from RICD specifically by reducing FASL upon TCR restimulation.

### C75 Treatment Protects Effector CD4 T Cells Derived From Multiple Memory T Cell Compartments

**Figure 7:**
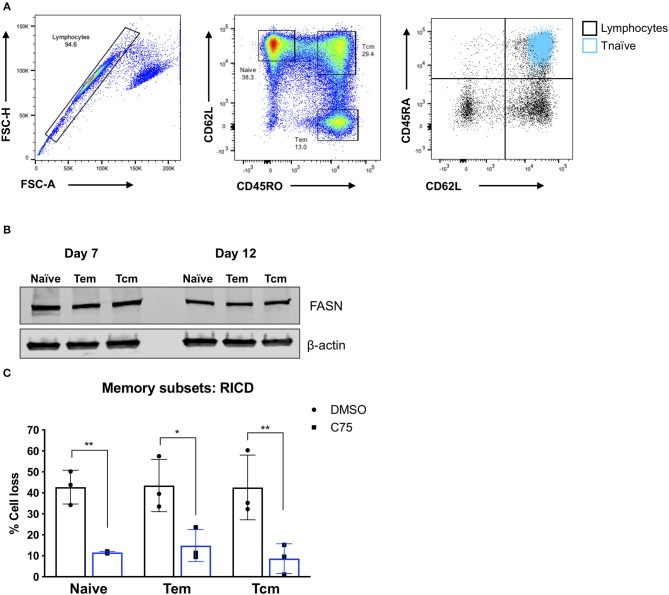
Effector T cells derived from memory T cell subsets are protected from RICD upon C75 treatment. **(A)** Sorting strategy. CD4 T cells were isolated from healthy donors and then subjected to FACS for naïve, effector memory (Tem), and central memory (Tcm) T cell populations. Naïve T cells were also confirmed to be CD45RA+ (right). **(B)** T cell subsets were sorted as in **(A)** and then activated with Immunocult. Whole cell lysates were collected on days 7 and 12 and subjected to immunoblot analysis for fatty acid synthase (FASN) expression. β-actin served as a loading control. **(C)** Effector T cells derived from the subsets as in **(A)** were treated with 15 μM C75 or a DMSO control for 1 h on day 12 post-activation and then restimulated with 100 ng/ml OKT3 to induce RICD. RICD was measured the next day be PI staining and flow cytometry. Statistical significance was determined by two-way ANOVA with Sidak's multiple comparisons test. ^**^p = 0.0061, ^*^p = 0.0107, ^**^p = 0.0033.

Although C75 treatment significantly protected bulk effector CD4 T cells from RICD (Figures 1B,C), we wondered whether this protection was specific to naïve T cell-derived effectors, or if memory T cell populations would also be rescued from RICD. To this end, we sorted naïve, effector memory (Tem), and central memory (Tcm) T cells from healthy human donors (Figure 7A). We first assessed expression levels of FASN in activated T cells from each subset on days 7 and 12 post-activation. FASN expression was comparable among naïve, Tem, and Tcm-derived effectors at each time point, although FASN expression was slightly higher at day 7 compared to day 12 (Figure 7B). Finally, CD4 T cells from each subset were treated with C75 on day 12 and then restimulated to assess RICD sensitivity. C75 treatment significantly protected all T cell subsets from RICD (Figure 7C). Collectively, our data suggests that effectors derived from naïve and memory T cell compartments show comparable FASN expression, and that the mechanism of C75 protection from RICD is conserved among T cell subsets.

### C75 Treatment Skews CD4 T Cells Toward a Th2 Phenotype

**Figure 8:**
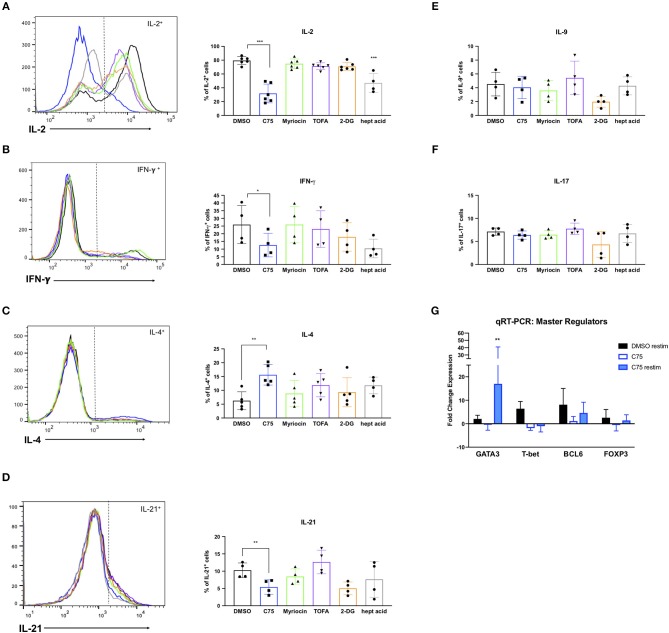
Metabolic inhibitor treatments impact Th1/Th2 polarization. CD4 T cells from healthy donors were treated with various inhibitors overnight on day 11 or 12 post-activation. Cells were restimulated with PMA/ionomycin for 4 h and subjected to intracellular cytokine staining for **(A)** IL-2, ^***^p < 0.0001 **(B)** IFN-γ, *p = 0.0415 **(C)** IL-4, **p = 0.0089. **(D)** IL-21, ^**^p = 0.0034 **(E)** IL-9, and **(F)** IL-17. The percentage of positive cells for each cytokine was quantified by flow cytometry and plotted for multiple donors. Statistical significance was determined by RM one-way ANOVA with Dunnett's multiple comparisons test. **(G)** CD4 T cells from healthy donors as in **(A–F)** were restimulated with PMA/ionomycin for 4 h and subjected to qRT-PCR. Expression data of each transcription factor was normalized to expression of the 18s rRNA housekeeping gene. All groups were then compared to DMSO-treated T cells that had not been restimulated to calculate fold changes. Statistical significance was determined by two-way ANOVA with Dunnett's multiple comparisons test. ^**^p = 0.0066.

RICD sensitivity in human CD4 T cells is governed in part by specific cytokine production. The first observation of this was published in mouse T cells, in which IL-2 exposure programed T cells to die by RICD (Lenardo, 1991). IL-2 exposure upregulates pro-apoptotic FASL in CD8 T cells (Dai et al., 1999). Additionally, IFN-γ was shown long ago to boost RICD sensitivity (Liu and Janeway, 1990; Refaeli et al., 2002). Overall, a Th1 effector phenotype is generally associated with higher death sensitivity compared to a Th2 phenotype, explained largely by differences in extrinsic, FAS-mediated apoptosis (Zhang et al., 1997). We therefore investigated the effect of overnight metabolic inhibitor treatments on the Th1/Th2 cytokine profile of our human CD4 T cells. To our surprise, C75 treatment resulted in a substantially lower proportion of IL-2-producing T cells compared to all other compounds tested (Figure 8A), consistent with the reduction of cells in S-phase observed previously (Figure 5A). The fraction of Th1-type cells producing IFN-γ trended lower in C75 treated cells (Figure 8B), whereas the proportion of Th2-type IL-4+ T cells was significantly increased (Figure 8C). The amount of IL-21-producing T follicular helper (Tfh)-like cells was also reduced with C75 treatment (Figure 8D), whereas IL-9- (Figure 8E) and IL-17-producing T cells (Figure 8F) were unaffected.

To further investigate the effect of C75 treatment on Th2 skewing, we measured expression levels of the Th2-type master transcription factor GATA3, along with other well-known master regulators such as T-bet (Th1), FOXP3 (Treg), and BCL6 (Tfh). Consistent with the Th2-skewing of cytokine production (Figures 8A–C), C75-treated cells also demonstrated a marked increase in GATA3 expression (Figure 8G) with no change in BCL6 or FOXP3 expression, and a trend toward lower T-bet expression. GATA3 expression was most significantly upregulated in C75-treated T cells that were restimulated, with an average of a 17-fold increase compared to controls (Figure 8G). Together, these observations suggest that FASN blockade using C75 is sufficient to skew human CD4 T cells to a Th2-like effector phenotype, which confers greater RICD resistance by limiting FASL induction upon TCR restimulation (Snow et al., 2010). Simultaneously, FASN inhibition may restrain the proportion of cycling CD4 T cells by limiting production of IL-2, another key driver of RICD sensitization.

## Discussion

Our studies provide novel evidence that inhibition of FASN protects primary human CD4 and CD8 effector T cells from RICD. This effect was specifically associated with blockade of FASN using C75, because inhibitors of other checkpoints in fatty acid metabolism such as TOFA, myriocin, and etomoxir did not reduce RICD sensitivity. FASN may be an attractive oncology drug target because it is an enzyme readily targeted by small molecule inhibitors, it is differentially expressed in tumor cells, and its sustained activity is needed for tumor growth and survival (Buckley et al., 2017). Given that effector T cell metabolism is analogous to rapidly dividing cancerous cells, we felt that it was important to examine the impact of this inhibitor (C75) in particular on T cell homeostasis. C75-mediated RICD resistance could enhance anti-tumor immunity by sparing more terminally-differentiated cytotoxic effector T cells from apoptosis. This could be potentially beneficial for emerging adoptive T cell therapies as well; e.g., chimeric antigen receptor (CAR) T cells could be rendered more durable upon pre-treatment with C75 in order to survive sustained repeated TCR stimulation. Indeed, CAR T cells have been shown to be sensitive to RICD upon adoptive transfer, via upregulation of FAS and FASL (Tschumi et al., 2018). However, it should also be noted that C75 treatment caused a large drop in ATP and IL-2 production, as well as a reduction in the amount of T cells cycling through S-phase. These data suggest that C75 could also abrogate robust T cell expansion if given early in the adaptive immune response. Additionally, we noted that C75 treatment skewed primary effector CD4 T cells toward a Th2-like phenotype, which could be detrimental to some immune responses, including anti-tumor T cell reactions that require IFN-γ. In addition, the low production of IL-2 may be contributing to protection from RICD, but could also make T cells more susceptible to CWID.

Our study joins many others that have underscored the importance of fatty acid metabolism in T cell function, highlighting the complexities of pharmacological and/or genetic alterations of this pathway. For example, CD4 T cell fatty acid metabolism was recently investigated in the differentiation and development of memory T cells in a *Plasmodium chabaudi* chronic malaria mouse model. Mice deficient in ACC1 formed fewer CD4 memory T cells, and an early shift to fatty acid synthesis was therefore identified as an important metabolic driver of CD4 memory (Ibitokou et al., 2018). When ACC1 was inhibited pharmacologically with TOFA, there was a significant reduction in the synthesis rate of fatty acids in activated CD4 T cells. C75 treatment in infected mice was lethal, however, presumably due to toxic accumulation of malonyl-CoA. We also noted differential effects with ACC vs. FASN inhibition in our RICD studies: whereas blockade of ACC with TOFA increased RICD levels, C75 treatment consistently decreased RICD sensitivity. Further work is required to determine if excess malonyl-CoA accumulation may contribute to RICD resistance in C75 treated T cells. Malonyl-CoA may facilitate important post-translational modifications, although it is not clear whether malonylation is dependent on the available pool of malonyl-CoA. Of note, malonylation of GAPDH impacted pro-inflammatory cytokine production in bone marrow-derived macrophages (BMDMs), modulating both its' enzymatic activity and RNA-binding capacity (Galvan-Pena et al., 2019). Although this has not been explored in human T cells, malonylation processes could be impacted by metabolic disruptions such as C75-mediated inhibition of FASN, and should be explored further.

The different outcomes of C75 and TOFA treatment could point to important differences between these steps in the fatty acid biosynthetic pathway, or could lend more credence to the off-target effects of C75. C75 has been shown to interfere with all three enzymatic functions of FASN (thioesterase, β-ketoacyl synthase, and enoyl reductase), giving it both potent and broad activity (Rendina and Cheng, 2005), including the ability to agonize CPT-1 activity (Chen et al., 2014). Importantly, siRNA-mediated knockdown of FASN also decreased RICD, although not as effectively. Other reported targets of C75 include the proapoptotic protein disulfide isomerase family A member 3 (PDIA3) (Cheng et al., 2014), and the mitochondrial β-ketoacyl-acyl carrier protein synthase (*Hs*mtKAS) (Chen et al., 2014). Pharmacologic inhibition of PDIA3 reduces apoptotic signaling by reducing BAK oligomerization (Zhao et al., 2015). Although we did not pursue this avenue, it is possible that C75 is also mediating RICD protection via PDIA3 inhibition. However, we believe the main mechanism of action is mediated via disruption of extrinsic rather than intrinsic apoptosis signals, given the robust decrease in FASL induction we observed. Additionally, C75 treatment in HEK293T cells was believed to impair mitochondrial function by *Hs*mtKAS disruption and a loss of downstream lipoic acid (LA) synthesis, because supplementation of LA restored mitochondrial function (Chen et al., 2014). Consistent with our Seahorse analyses, C75 treatment also disrupted mitochondrial function in CD4 T cells, which could have been due to interactions with *Hs*mtKAS. Interestingly, Chen et al. noted that siRNA knockdown of FASN did not reduce mitochondrial function, but *Hs*mtKAS knockdown did. Given that our FASN knockdown did not reduce RICD to the extent of C75 treatment, it remains possible that *Hs*mtKAS could be contributing to RICD as well.

C75 is not the only example of a small molecule inhibitor that has created difficulty in interpreting T cell fatty acid metabolism findings. For example, long-chain FAO was thought to support regulatory T cell (Treg) and memory T cell development and survival, based on data largely from etomoxir-mediated inhibition of CPT1. However, recent work from the Berod lab has shown that genetic targeting of CPT1 yielded different conclusions, and posited that etomoxir mediates off-target effects that are actually responsible for differences in T cell differentiation and function (Raud et al., 2018). Hence pharmacological approaches for altering T cell metabolism must be weighed carefully, as demonstrated by the divergent data from studies of fatty acid synthesis blockade. It should also be noted that our *in vitro* system of RICD studies does not discern between the free fatty acids available to T cells in culture media, which has considerably altered the results of T cell studies from other groups (Eleftheriadis et al., 2016). Our future studies will investigate how exogenous fatty acids and culture media conditions may affect RICD sensitivity of human T cells *in vitro*.

Despite these limitations, experimental manipulations of fatty acid metabolism have yielded important insights into its key role in shaping CD4 T cell differentiation. In another study, memory CD4 T cells isolated from healthy donors and activated for 5 days in the presence of TOFA, C75, or cerulenin all exhibited a reduced percentage of IL-17+ cells but normal IFN-γ+ cells, suggesting that inhibiting fatty acid synthesis impacts Th17 cells more than Th1 cells (Cluxton et al., 2019). In our *in vitro* system, C75 treatment skewed CD4 effector T cells to a Th2-like phenotype with a higher percentage of GATA3+ IL-4 producing cells and an associated trend in decreased IFN-γ production. In agreement with these findings, deletion of ACC1 in murine T cells increased susceptibility to *Mycobacterium bovis* BCG infection, which was due in part to decreased IFN-γ+ CD4 T cells (Stuve et al., 2018). Collectively, these results would suggest that *de novo* fatty acid synthesis does contribute to Th1 effector cytokine production in effector CD4 T cells, which could be regulated differently in memory CD4 T cells. Nonetheless, C75 treatment reduced RICD in CD4 effector T cells from both naïve and memory compartments. We did not see a significant decrease in IFN-γ+ CD4 T cells with TOFA treatment, which further highlights possible discrepancies in blocking FASN vs. ACC. Importantly, our findings linking C75-mediated RICD resistance to skewed Th cell phenotypes are in agreement with older RICD literature; the cytokines that sensitize T cells to die by RICD (IL-2 and IFN-γ) were both downregulated, whereas IL-4 was upregulated. Variable RICD sensitivity in Th effector subsets was ultimately linked to differences in FAS-mediated apoptosis via FASL upon TCR restimulation (Varadhachary et al., 1997; Zhang et al., 1997; Dai et al., 1999; Cencioni et al., 2015), which was also significantly reduced in C75 treated CD4 T cells. IL-17 is also associated with protection from RICD by downregulation of FASL (Kim et al., 2013), but we did not see a change in IL-17 production in any of the treatment groups.

Although 2-DG treatment did not reduce RICD in CD4 T cells, the specific GAPDH inhibitor heptelidic acid did. We also noted that that although C75 treatment attenuated glycolytic flux, it did not directly impede GAPDH activity. Furthermore, the reduction in glycolytic flux achieved with C75 is likely not responsible for impaired RICD, since it was comparable to 2-DG in comparative Seahorse analyses. Intriguingly, GAPDH has multiple “moonlighting” functions in addition to its' role in glycolysis, which we did not explore here. For example, GAPDH can bind to adenylate-uridylate (AU)-rich elements in the 3′-UTR of IFN-γ and IL-2 mRNAs (Nagy and Rigby, 1995), and directly modulate their expression in CD4 T cells when glucose availability is restricted (Chang et al., 2013). GAPDH can also localize to diverse cellular compartments under certain conditions, such as oxidative stress (Tristan et al., 2011), supporting the notion that GAPDH moonlighting functions could be responsible for the decrease in RICD sensitivity we observed in CD4 T cells after heptelidic acid treatment.

Unlike CD4 T cells, we previously showed that 2-DG treatment could decrease RICD of CD8 effector T cells (Larsen et al., 2017). However, it is important to note that CD8 T cells are substantially more sensitive to RICD than their CD4 counterparts (compare Figure 1B to Supplemental Figure 1A). This could be linked to more rapid cell division *in vitro* and a greater reliance on glycolysis in CD8 effector T cells. Indeed, RICD was more significantly reduced in CD8 T cells by culturing in galactose vs. glucose, which effectively prevents glycolysis altogether (8). Acetyl-CoA carboxylase 2 (ACC2) was shown to be dispensable for CD8 T cell mediated immunity (Lee et al., 2015), which implies a heavier reliance on glycolysis vs. FAO in effector CD8 T cells. On the other hand, vaccine-elicited CD8 T cells were able to proliferate independently of aerobic glycolysis in a murine model (Klarquist et al., 2018). Interestingly, blocking glycolysis with 2-DG *in vivo* did not impede vaccine-elicited T cell expansion, but did reduce CD8 T cell responses to live infection. Therefore, the precise conditions of T cell activation, including the abundance of antigen and inflammatory “danger” signals, can result in fundamentally different outcomes for T cell metabolic reprogramming, downstream cytokine production, and apoptosis sensitivity.

Rather than considering glycolysis and FAO as opposing ATP-generating programs, perhaps the ultimate underlying governor of RICD sensitivity is the basal metabolic state of the T cell. Our bioenergetics analyses revealed that C75 significantly lowered ATP production from the mitochondria, whereas TOFA treatment increased proton leak, which can indicate mitochondrial damage. Basal respiration was also significantly decreased in C75-treated T cells. Therefore, relative ATP production and basal respiration should be investigated further as potential predictors of RICD sensitivity in effector T cells. For example, FAS-mediated death during RICD may be ATP-dependent, although we were not able to directly test this hypothesis due to the technical difficulty of replenishing ATP stores to C75-treated T cells. In conclusion, our findings indicate that FASN inhibition substantially lowers the basal metabolism of CD4 T cells. This drop in anabolic metabolism results in decreased IL-2 production and slowed cell cycling, Th2 skewing, impaired TCR-induced FASL expression, resulting in reduced RICD sensitivity.

## Materials and Methods

### T Cell Isolation and Culture Conditions

Peripheral blood mononuclear cells (PBMC) were obtained from healthy human donors at the National Institutes of Health (NIH) Blood Bank. Access to anonymous Blood Bank donors was kindly provided by Dr. Michael Lenardo. CD4 T cells were purified from PBMC by immunomagnetic negative selection using the EasySep Human CD4 T cell isolation kit (Stem Cell Technologies). Purity was routinely assessed by flow cytometry and was ~95% (Supplemental Figure 1A). Primary T cells were activated with ImmunoCult Human CD3/CD28/CD2 T Cell Activator (Stem Cell Technologies), according to the manufacturer's instructions in complete RPMI (RPMI 1640 (ThermoFisher Scientific) + 10% fetal calf serum (FCS) (Sigma Aldrich) + 1% penicillin/streptomycin (Lonza). After 3 days, activated cells were washed with PBS and subsequently cultured in complete RPMI with 100 U/mL rIL-2 (PeproTech).

### siRNA-Mediated Knockdowns in Primary T Cells

T cells were electroporated with siRNAs against FASN (ThermoFisher assay ID s5031) or Stealth RNAi negative control (medium GC content) siRNA (ThermoFisher) as a non-specific (NS) control using the Amaxa Nucleofection 4D system and the P3 Primary Cell kit (Lonza). Assays were conducted 4 days post-electroporation for peak knockdown efficiency. Knockdown efficiencies were assessed by immunoblotting for every experiment.

### Restimulation-Induced Cell Death (RICD) and FAS Ligation Death Assays

RICD assays were conducted as described previously (Katz and Snow, 2013). Briefly, 1 × 10^5^ T cells in 0.2 ml complete RPMI were restimulated with anti-CD3 mAb (clone OKT3) in triplicate wells of a 96 well round-bottom plate for 24 h. Cells were stained with 10 μl of propidium iodide (PI, 1 μg/ml stock) (ThermoFisher) to distinguish live and dead cells and immediately analyzed on a BD Accuri C6 flow cytometer. Death was quantified as percent cell loss, based on quantification of viable cells collected under constant time, where % cell loss = (1 – [number of viable cells (treated)/number of viable cells (untreated)]) × 100. For small molecule inhibitors, T cells were pre-treated overnight with 15 μM C75, 5 μM myriocin, 10 μM etomoxir, 10 μM (TOFA), 2 mM 2-deoxy-D-glucose (2-DG), 0.25 μM heptelidic acid (hept acid), 5 mM CAY10703 or a DMSO solvent control prior to OKT3 restimulation. FAS death assays were conducted similar to RICD assays, however cells were stimulated for 24 h with agonistic anti-FAS Ab (APO1.3) + 1 μg/mL Protein A (Sigma Aldrich). Soluble Fas ligand was quantified from T cell supernatants in triplicate with the Human Fas Ligand/TNFSF6 Quantikine ELISA kit (R&D Systems). Pre-treated T cells were restimulated for 4 h with 200 ng/ml OKT3 and supernatants were cleared by 5 min centrifugation at 1,400 rpm.

### Flow Cytometry and FACS of Memory T Cell Populations

For intracellular cytokine staining, T cells were pre-treated overnight with various metabolic inhibitors and then treated for 30 min with Brefeldin A (BD Biosciences). T cells were then stimulated with 1 μg/ml ionomycin and 20 ng/ml PMA for 4 h, fixed with 1.5% paraformaldehyde and permeabilized with cold methanol. Antibody staining was conducted at room temperature for 30 min in FACS buffer (PBS + 1% BSA + 0.02% sodium azide) with either anti-IL-2 APC (ThermoFisher MQ1-17H12), anti-IL-4 APC (ThermoFisher 8D4-8), anti-IFN-γ PE (R&D Systems IC285P), anti-IL-21 APC (Biolegend 513008), anti-IL-9 PE (Biolegend 507605), and anti-IL-17A FITC (Biolegend 512304). Cell surface Antibody staining was conducted on ice for 30 min in FACS buffer with CD36-FITC (Biolegend 336204) or an isotype control.

For PI cell cycle analysis, 1 × 10^6^ T cells were washed in cold PBS and resuspended in 0.5 ml of propidium iodide (PI) stain solution: 50 μg/ml PI + 1 mg/ml RNAse A (Sigma) + 0.1% Triton X-100 + 1 mg/ml sodium citrate in PBS for 30 min on ice. Cells in G1, S, and G2/M phases of the cell cycle were gated and quantified by flow cytometry (linear scale). Active caspase-3 staining was conducted with the PE Active Caspse-3 Apoptosis kit according to the manufacturer's instructions (BD Biosciences 550914). Day 12 CD4 T cells were pre-treated for 1 h with DMSO or C75, and then were restimulated overnight with 200 ng/ml OKT3.

Fluorescence-activated cell sorting (FACS) was conducted on isolated human CD4 T cells labeled with CD45RO-APC (Biolegend 304210), CD62L-PE (Biolegend 304806), and CD45RA-FITC (Biolegend 304106) using the FACSAria Fusion (BD). All flow cytometry analysis was conducted using FlowJo v10 software.

### Western Blotting

For general immunoblotting, cells were lysed in 1% Nonidet P-40 (NP-40) lysis buffer (50 mM Tris [pH 7.4], 150 mM NaCl, 0.5 mM EDTA, 1% NP-40, 0.5% sodium deoxycholate, 1 mM Na_3_VO_4_, 1 mM NaF) + complete protease inhibitors (Roche) for 30 min on ice. Lysates were cleared by centrifugation, boiled in 2x sample buffer (Laemmli buffer + 50 μM 2-βME) and separated on 4–20% SDS-PAGE gels (Bio-Rad). Proteins were transferred to nitrocellulose membranes for 10 min using the TransBlot Turbo system (Bio-Rad) and subsequently blocked with Odyssey blocking buffer (LI-COR). Blots were probed with the following Abs: anti-FASN (Cell Signaling Technology #3180), anti-MCL-1 (Cell Signaling Technology #94296), anti-BCL-2 (Cell Signaling Technology #4223), anti-FASL (BD Pharminogen #556372), anti-BIM (Enzo Life Sciences), anti-NUR77 (Biolegend, clone 1E10A15) and anti-β-actin (Sigma-Aldrich). Blots were incubated with IRDye anti-Rabbit or anti-Mouse secondary antibodies (LI-COR) and imaged using the Odyssey CLx instrument. Band intensity quantification was conducted with StudioLite Software (LI-COR).

### Metabolic Flux Analysis

Human CD4 T cells were expanded in culture for 10–12 days and pre-treated overnight with various inhibitors. The next day, cells were counted via trypan blue and seeded at 125,000 cells/well in Seahorse XF96 Cell culture microplates, which were pre-treated with 22.4 μg/ml Cell-Tak solution for 20 min (Corning). Mito Stress tests were conducted according to the manufacturer's instructions (Agilent) with Seahorse Agilent pH7.4 media + 1 mM pyruvate + 10 mM glucose + 2 mM L-glutamine. Final concentrations of inhibitors after injections were 1.5 μM oligomycin A, 1.5 μM trifluoromethoxy carbonylcyanide phenylhydrazone (FCCP) (Sigma Aldrich), 0.5 μM rotenone and 0.5 μM antimycin A.

GAPDH activity was measured using the colorimetric Glyceraldehyde 2 Phosphate Dehydrogenase Activity Assay Kit (Abcam). 4 × 10^6^ CD4 T cells were treated overnight with the various inhibitors or DMSO control, washed in cold PBS, and lysed in 150 μl of GAPDH buffer. Samples were measured in triplicate wells and the average value was plotted for each donor.

### Quantitative RT-PCR on CD4 T Cells

Human CD4 T cells were expanded in culture for 10–12 days and pre-treated overnight with DMSO or 15 μM C75. RNA was isolated from 2 × 10^6^ CD4 T cells by Direct-zol RNA mini kit (Zymo Research) and then subjected to the iScript Advanced cDNA synthesis kit (Bio-Rad). Cycle conditions: 46°C for 20 min, 95°C for 1 min, 4°C. qRT-PCR reactions were prepared using SsoAdvanced Universal SYBR Green Supermix (Bio-Rad). Cycling conditions 95°C for 30 s [95°C for 15 s, 60°C for 30 s] × 39, 95°C for 5 s followed by melt curve analysis.

The primers used were GATA-3 Forward: 5′-TGTCTGCAGCCAGGAGAGC-3′, Reverse: 5′-ATGCATCAAACAACTGTGGCCA-3′. T-bet Forward: 5′-CCCCCAAGGAATTGACAGTTG-3′, Reverse: 5′-GGGAAACTAAAGCTCACAAAC-3′ FOXP3 Forward: 5′-GATGGTACAGTCTCTGGAGC-3′, Reverse: 5′-GGGAATGTGCTGTTTCCATGG-3′.

18s Forward: 5′-ACCCGTTGAACCCCATTCGTGA-3′, Reverse: 5′-GCCTCACTAAACCATCCAATCGG-3′.

BCL6 Forward: 5′-CATGCAGAGATGTGCCTCCACA-3′,Reverse: 5′-TCAGAGAAGCGGCAGTCACACT-3′.

### Statistics

Statistical analyses were conducted using GraphPad Prism 8.0.1 software. Error bars in all figures indicate ± standard deviation. All statistical tests performed are indicated in the figure legends. For RICD assays, each data point represents the average % cell loss from an individual donor (the average of triplicate samples). We performed repeated measures (RM) one-way ANOVA tests with or without Dunnett's multiple comparisons test as needed for multiple comparisons. Statistical tests were reviewed by Dr. Cara H Olsen (Director, Uniformed Services University Biostatistics Consulting Center), who kindly provided guidance on appropriate tools.

## Data Availability Statement

The datasets generated for this study are available on request to the corresponding author.

## Author Contributions

KV designed the experiments, conducted and analyzed experiments, and wrote the manuscript. CL assisted with conducting intracellular flow cytometry and conducted all qRT-PCR experiments and analyses. KP assisted with flow cytometry. AS supervised the project and provided edits.

### Conflict of Interest

The authors declare that the research was conducted in the absence of any commercial or financial relationships that could be construed as a potential conflict of interest.

## Supplementary Material

Supplemental Figure 1 CD36 expression, etomoxir dose curve, and mitochondrial mass assessment in activated CD4 T cells. **(A)** CD4 T cells from healthy donor PBMCs were isolated by negative selection and checked for purity by flow cytometry. Average purity was ~95%. **(B)** CD4 T cells from healthy donors were treated with DMSO or C75 overnight after 12 days post-activation. CD36 expression was measured by flow cytometry. MFI, mean fluorescence intensity. **(C)** Day 12 CD4 T cells were treated overnight with DMSO or etomoxir at the indicated doses. Cells were then restimulated with 100 ng/ml OKT3 and RICD was measured by PI staining. Each data point represents the average % cell loss from an individual donor. RM one-way ANOVA with Dunnett's multiple comparisons test ^*^*p* = 0.0203. **(D)** Day 12 CD4 T cells were treated overnight with various inhibitors or a DMSO control. Mitochondria were labeled with Mitotracker Green and mitochondrial mass was measured by flow cytometry. Values indicate mean fluorescence intensity of Mitotracker from one representative experiment (*N* = 3).

Supplemental Figure 2 FASN inhibition protects human CD8 T cells from RICD and reduces ATP and glycolysis. **(A)** CD8 T cells from healthy donors were treated with various inhibitors overnight after 12 days post-activation and then restimulated with 100 ng/ml OKT3 for 24 h. Restimulation-induced cell death (RICD) was measured by propidium iodide (PI) staining. Each data point represents the average % cell loss from an individual donor. One-way ANOVA with Dunnett's multiple comparisons test ^***^*p* < 0.0001. **(B)** CD8 T cells were pre-treated with C75 for 1.5 h before restimulation with 100 ng/ml OKT3. Multiple *t*-tests were performed [1 ng/ml] ^*^*p* = 0.0434, [5 ng/ml] ^*^*p* = 0.0438, [25 ng/ml] ^**^*p* = 0.0099, [50 ng/ml] ^**^*p* = 0.0073.
