# Supplementary figures and images for: Fatty Acid Synthase Contributes to Restimulation-Induced Cell Death of Human CD4 T Cells
Source: Front Mol Biosci. 2019 Oct 15;6:106. doi: 10.3389/fmolb.2019.00106 (PMC6803432; doi:10.3389/fmolb.2019.00106)

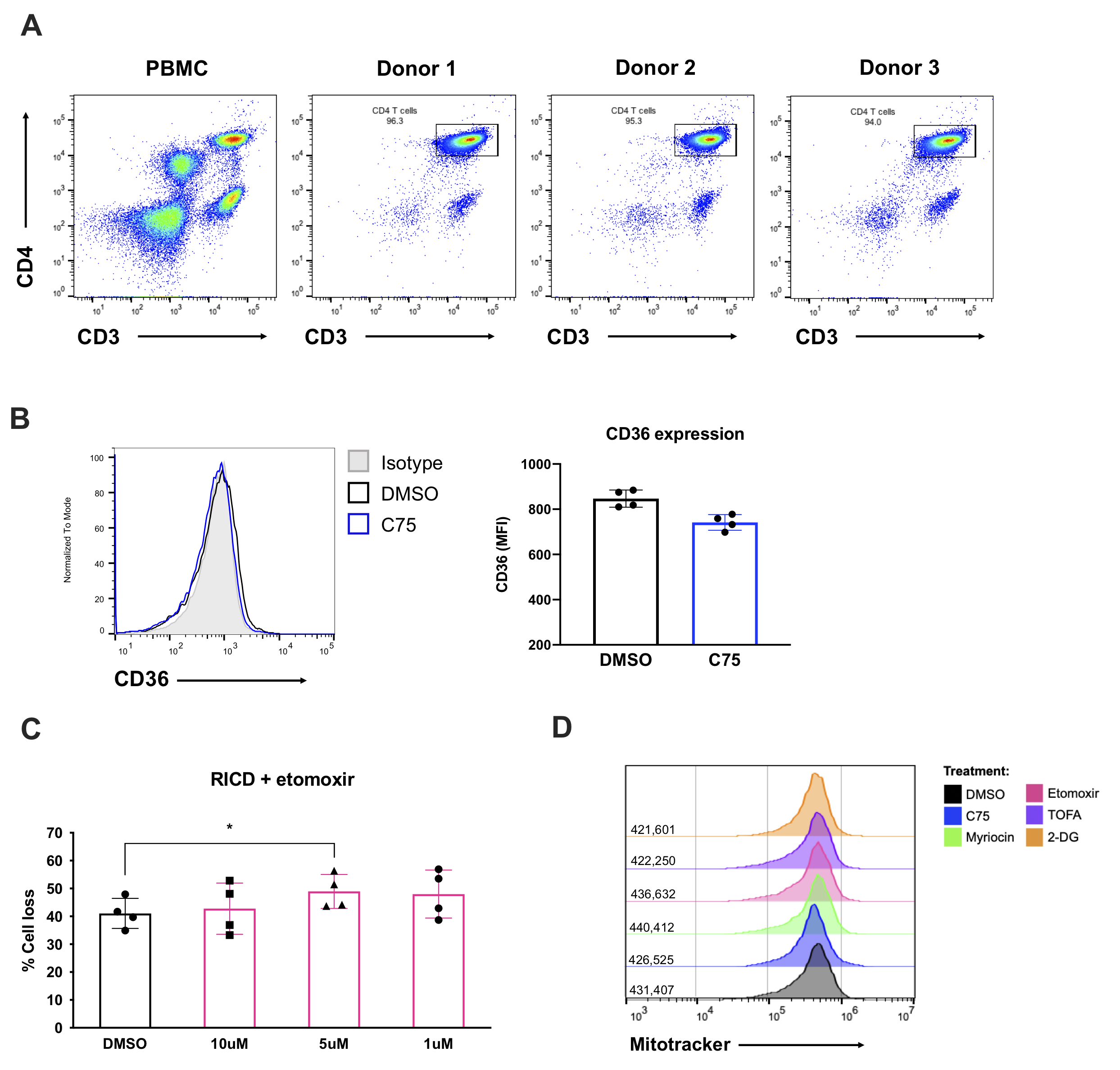

Supplement: Supplemental Figure 1 — CD36 expression, etomoxir dose curve, and mitochondrial mass assessment in activated CD4 T cells. (A) CD4 T cells from healthy donor PBMCs were isolated by negative selection and checked for purity by flow cytometry. Average purity was ~95%. (B) CD4 T cells from healthy donors were treated with DMSO or C75 overnight after 12 days post-activation. CD36 expression was measured by flow cytometry. MFI, mean fluorescence intensity. (C) Day 12 CD4 T cells were treated overnight with DMSO or etomoxir at the indicated doses. Cells were then restimulated with 100 ng/ml OKT3 and RICD was measured by PI staining. Each data point represents the average % cell loss from an individual donor. RM one-way ANOVA with Dunnett's multiple comparisons test *p = 0.0203. (D) Day 12 CD4 T cells were treated overnight with various inhibitors or a DMSO control. Mitochondria were labeled with Mitotracker Green and mitochondrial mass was measured by flow cytometry. Values indicate mean fluorescence intensity of Mitotracker from one representative experiment (N = 3). [file Image_1.TIFF]

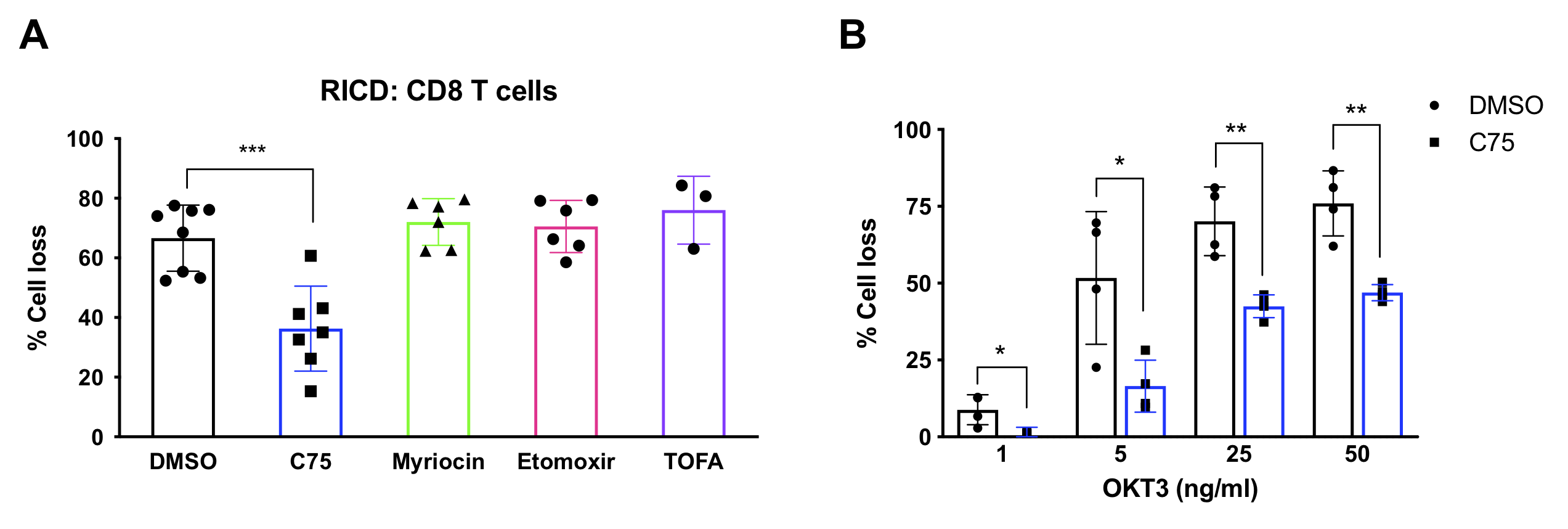

Supplement: Supplemental Figure 2 — FASN inhibition protects human CD8 T cells from RICD and reduces ATP and glycolysis. (A) CD8 T cells from healthy donors were treated with various inhibitors overnight after 12 days post-activation and then restimulated with 100 ng/ml OKT3 for 24 h. Restimulation-induced cell death (RICD) was measured by propidium iodide (PI) staining. Each data point represents the average % cell loss from an individual donor. One-way ANOVA with Dunnett's multiple comparisons test ***p < 0.0001. (B) CD8 T cells were pre-treated with C75 for 1.5 h before restimulation with 100 ng/ml OKT3. Multiple t-tests were performed [1 ng/ml] *p = 0.0434, [5 ng/ml] *p = 0.0438, [25 ng/ml] **p = 0.0099, [50 ng/ml] **p = 0.0073. [file Image_2.TIFF]
